# Whole-Genome Sequencing Unveils the Uniqueness of Yushu Yaks (*Bos grunniens*)

**DOI:** 10.3390/ijms26083879

**Published:** 2025-04-19

**Authors:** Bao Cai, Xiaoyun Wu, Yilin Shi, Yandong Kang, Ziqiang Ding, Shaoke Guo, Mengli Cao, Liyan Hu, Ben Zhang, Xingdong Wang, Jie Pei, Qianyun Ge, Lin Xiong, Songshan Zhang, Xian Guo

**Affiliations:** 1 Key Laboratory of Yak Breeding of Gansu Province, Lanzhou Institute of Husbandry and Pharmaceutical Sciences, Chinese Academy of Agricultural Sciences, Lanzhou 730050, China; 82101235499@caas.cn (B.C.); wuxiaoyun@caas.cn (X.W.); shi_yilin37@163.com (Y.S.); kangyandong0901@163.com (Y.K.); dingziqiang1997@163.com (Z.D.); gsk1125@163.com (S.G.); caomengliaaa@163.com (M.C.); huliyan2020@163.com (L.H.); zhangbencaas@163.com (B.Z.); wxd17339929758@163.com (X.W.); peijie@caas.cn (J.P.); geqianyun@caas.cn (Q.G.); xionglin@caas.cn (L.X.); 2 Beijing Institute of Animal Husbandry and Veterinary Medicine, Chinese Academy of Agricultural Sciences, Beijing 100080, China

**Keywords:** Yushu yak, whole-genome resequencing, population structure, selection signals

## Abstract

The Yushu yak is one of China’s distinctive yak breeds, primarily distributed in the Yushu Tibetan Autonomous Prefecture of Qinghai Province and its surrounding areas. Yushu yaks are not only economically and culturally significant but also play a crucial role in protecting the ecosystem of the Qinghai-Tibet Plateau and promoting sustainable development. However, there are no clear records regarding the ancestry, population structure, and unique traits of Yushu yaks. Therefore, this study conducted an analysis of genetic diversity, population structure, and selection signals in Yushu yak populations, aiming to provide references for the conservation and utilization of the breed genetic resources. The results of the analysis showed that the Yushu yak population has high genetic diversity and low inbreeding coefficients, indicating a stable genetic structure. Population structure analysis revealed that the Yushu yak lineage is unique, with limited gene flow between domestic and wild yaks. Functional enrichment analysis of positively selected genes in Yushu yaks indicated prominent selection features related to growth and development as well as energy metabolism. Additionally, we classified the Yushu yak breeding bulls into family lineages based on kinship, which is essential for improving the efficiency of utilizing genetic resources and scientifically managing the population.

## 1. Introduction

**Figure 1:**
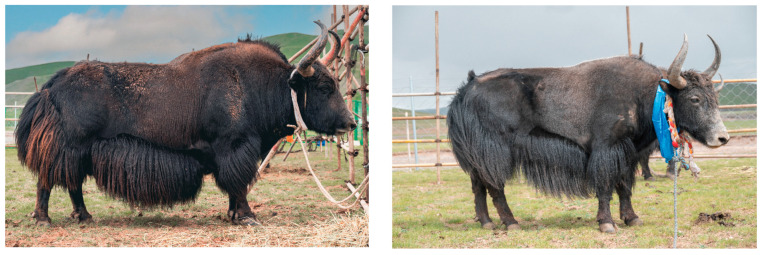
Physical appearance of Yushu yak breeding bulls.

The yak (*Bos grunniens*) is a unique livestock species adapted to cold, high-altitude pastures, primarily found in the Tibetan Plateau, Pamir Plateau, and surrounding regions. It exhibits remarkable adaptability to harsh climatic conditions. The production model of yaks is predominantly based on grazing, with their milk and meat regarded as green, organic food sources [1,2]. Moreover, yaks play a crucial role in maintaining and regulating the grassland ecosystem of the Qinghai-Tibet Plateau and in protecting biodiversity. Previous studies have shown that domestic yaks evolved from wild yaks and subsequently developed unique adaptations in different distribution areas [3,4,5]. The Yushu yak is one of China’s distinctive yak breeds, primarily found in the Yushu Tibetan Autonomous Prefecture of Qinghai Province and its surrounding areas. Yushu yaks have long lived in natural environments above 3700 m in altitude, characterized by cold climates and long periods of withered grass. They can utilize high-altitude pastures where other livestock cannot survive, exhibiting greater adaptability to low-pressure, thin-air conditions and cold alpine meadows (Figure 1). However, there are no clear records regarding the ancestry, population structure, and unique traits of Yushu yaks. As a distinctive livestock breed of the Qinghai-Tibet Plateau, the genetic diversity and specific genetic variations of Yushu yaks are critical for maintaining population adaptability, disease resistance, and resilience to environmental changes. Additionally, Yushu yak breeding bulls are valuable resources for genetic improvement. Protecting and preserving the superior genetic resources of these breeding bulls ensures the rational conservation and utilization of the breed’s gene pool, preventing the loss of precious genetic characteristics.

With the continuous advancement and development of high-throughput sequencing technology, genomic sequencing has become a crucial tool for uncovering genetic variation in organisms. Compared to traditional molecular biology techniques, whole-genome sequencing offers higher accuracy and can detect smaller variations and mutations. This provides more precise data for studying genetic differences between individuals, association analysis, adaptability research, and more [6,7]. Numerous studies have utilized whole-genome sequencing to analyze blood samples from cattle, thereby constructing genetic maps and evaluating population genetic diversity [8,9]. Additionally, evolutionary analyses and selective signal detection have been employed to investigate the population’s evolutionary history and environmental adaptation [10,11,12]. For example, Liu et al. [13] performed whole-genome sequencing on blood samples from wild yaks, domestic yaks, and yellow cattle, constructing a genetic map and discovering that yaks underwent natural selection and interspecies introgression during their evolution. Guo et al. [14] conducted whole-genome sequencing and selective sweep analyses on blood samples from Subei yaks and eight domestic yak breeds, identifying candidate genes such as *ACSF3*, *HMGCS1*, *MC1R*, *RFX5*, *CGMP*, and *PDE2A*, which are associated with immune response and growth development in Subei yaks. Jiang et al. [15] used GWAS (genome-wide association study) to identify SNPs related to yak body weight.

Whole-genome sequencing technology holds significant importance for the genetic improvement of livestock breeds. By analyzing genomic characteristics, it aids in identifying genes associated with important economic traits and adaptability in animals, thereby understanding the potential genetic basis underlying their unique traits [16]. Through evaluating the adaptability and family structure of Yushu yak breeding bulls, it is possible to effectively identify individuals with higher genetic value, providing decision support for population management. This is crucial for optimizing population management, promoting genetic improvement, and protecting genetic diversity. Analyzing the genetic diversity and population structure of Yushu yaks can reveal the extent of genomic variation and lineage origins, thus providing a scientific basis for the genetic characteristics of the population. Therefore, this study first assessed the genetic diversity and kinship of 18 Yushu yak breeding bulls and then classified them into families based on genetic distances. Subsequently, we conducted genetic background and adaptability analyses of the Yushu yak population. These efforts not only provide data support for current breeding and conservation efforts of Yushu yaks but also offer long-term guidance for future breed improvement and the rational utilization of resources.

## 2. Results

### 2.1. Construction of Genetic Map

**Figure 2:**
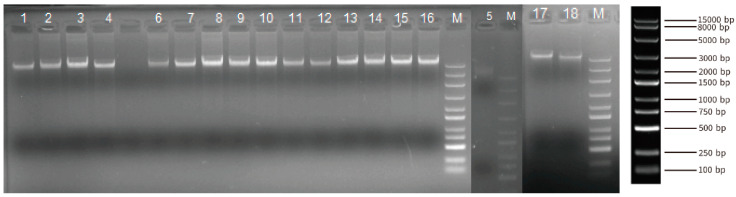
Composite Image of Agarose gel electrophoresis bands of genomic DNA from Three Unique Gels. (Each sample was loaded with 100 µL, with “M” denoting the marker. From top to bottom, the sizes are as follows: 15,000 bp, 8000 bp, 5000 bp, 3000 bp, 2000 bp, 1500 bp, 1000 bp, 750 bp, 500 bp, 250 bp, and 100 bp).

A total of 18 blood DNA samples from Yushu yaks were collected to conduct the whole-genome resequencing. The DNA samples from Yushu yaks exhibit uniform brightness, clear bands, and the absence of smearing by agarose gel electrophoresis (Figure 2). These results indicated that the quality of the genomic DNA samples is satisfactory for subsequent library preparation and sequencing (Table S1). We obtained 6,972,566,582 reads in these 18 individuals. Utilizing BWA software (v0.7.18), the sequencing reads were aligned to the yak genome, achieving a coverage of at least 96% of the genomic regions for each individual, with an average sequencing depth of 23.7× (Table S2).

**Figure 3:**
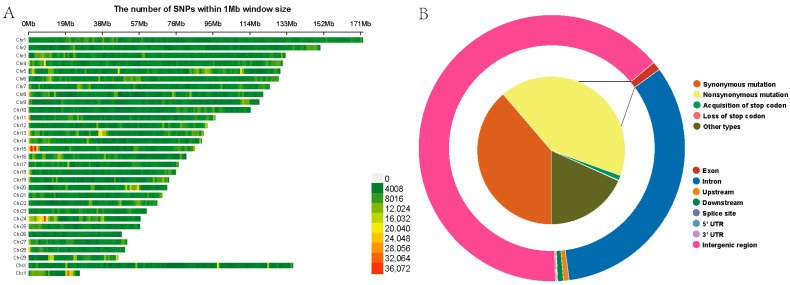
Construction of genetic map. (**A**) SNP density plot. (**B**) Functional annotation results of SNP regions.

Following variant detection and quality control, a total of 23,579,526 high-quality SNPs were identified among the 18 Yushu yaks, with 264,934 SNPs not mapped to any chromosome. The highest number of SNPs was found on chromosome 1, comprising 1,341,216 SNPs, while chromosome 26 had the fewest, with 355,293 SNPs (Table S3). To provide a clearer depiction of the SNP distribution across the chromosomes, we have constructed a heatmap illustrating the SNP density distribution on the chromosomes (Figure 3A). Gene function annotation results indicated that SNPs in the Yushu yak genome are predominantly located in intergenic regions (15,164,159 SNPs, accounting for 64.31%) and intronic regions (7,428,273 SNPs, representing 31.50%). The proportions of synonymous and nonsynonymous mutations were relatively low, consisting of 97,277 and 104,904 SNPs (Figure 3B, Table S4). The transition-to-transversion ratio (Ts/Tv) for the entire genome’s SNPs was calculated to be 2.21.

After analyzing the whole-genome copy number variations (CNVRs) in Yushu yaks, a total of 25,775 CNVRs were detected, with an aggregate length of 23,429,066 bp and an average length of 6274 bp, constituting 0.88% of the yak genome. This includes 14,969 deletions, 10,654 duplications, and 152 complex variations. Following the identification of structural variations (SVs) in Yushu yaks, 64,264 SVs were detected, comprising 24,829 deletions, 8002 insertions, 25,633 translocations, and 5800 duplications. Gene function annotation results indicate that SVs in the Yushu yak genome are predominantly located in intergenic regions (10,310, accounting for 61.52%) and intronic regions (5603, representing 33.43%) (Table S5).

### 2.2. Genetic Diversity Analysis of Yushu Yaks

**Figure 4:**
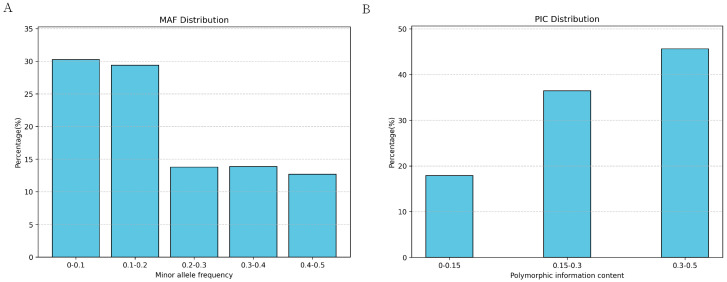
Distribution of genetic polymorphism. (**A**) Distribution of minimum allele frequency. (**B**) Distribution of polymorphic information content.

To investigate the genetic diversity within the Yushu yak population, we calculated various parameters based on SNP data, including polymorphic information content (PIC), heterozygosity, minimum allele frequency (MAF), and the number of runs of homozygosity (ROH). The results indicate that the average MAF of Yushu yaks is 0.2033. Within the distribution range of MAF, the largest proportion falls between 0–0.1 (30.28%), while the smallest proportion is found in the 0.4–0.5 range (12.67%) (Figure 4A). The polymorphic information content (PIC) of SNP loci in Yushu yaks ranges from 0.095 to 0.500, with an average PIC of 0.2869 (Figure 4B).

Further analysis indicated that the average observed heterozygosity (Ho) was 0.3074, and the average expected heterozygosity (He) was 0.2869 (Table 1). The average observed heterozygosity in the Yushu yak population exceeded the expected heterozygosity, suggesting a degree of differentiation within the group. Among the 18 Yushu yaks, we identified a total of 20,354 ROHs, with an average length of 148.68 Kb. The number of ROHs per individual varied from 914 to 1392, averaging 1130, with an average length of 168.12 Mb, indicating considerable individual variation. In terms of ROH distribution across chromosomes, the first chromosome exhibited the highest number of ROHs at 1240, while the 27th chromosome had the fewest at 282 (Table S6). Additionally, we calculated the inbreeding coefficient for each individual based on ROH, revealing an average fraction of the genome that is runs of homozygosity (FROH) of 0.0632 ± 0.002 for the Yushu yak population, indicating a relatively low level of inbreeding (Table S7).

### 2.3. Analysis of Intra-Population Kinship and Pedigree Construction in Yushu Yaks

**Figure 5:**
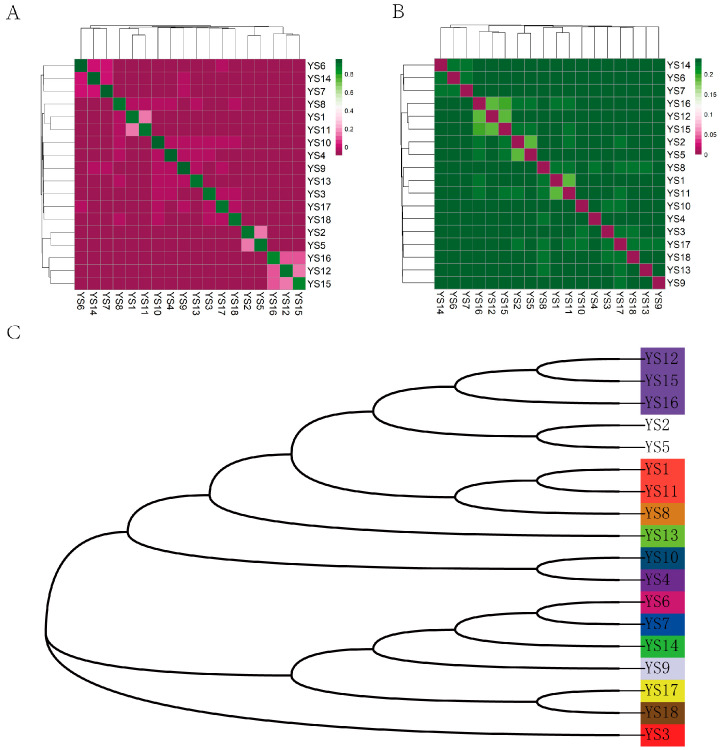
Kinship analysis. (**A**) Genetic distance matrix: this matrix illustrates the genetic distances between individuals within the population. (**B**) Kinship matrix: the kinship matrix depicts the pairwise genetic relationships among individuals. (**C**) Intra-population pedigree classification: This classification shows the familial relationships and lineage structures within the population. It categorizes individuals based on their genetic distances and kinship, with each color representing a distinct family lineage. The 18 Yushu yak breeding bulls were classified into 14 family lineages.

The results of the IBS distance matrix indicate that the IBS genetic distance within the Yushu yak population ranges from 0.185023 to 0.239055, with an average genetic distance of 0.244011 (Figure 5A,B). The visualization of the IBS distance matrix for the entire population aligns with the results of the G matrix analysis, indicating that most individuals of Yushu yaks share relatively distant genetic relationships, while a select few exhibit closer familial ties. Based on a kinship coefficient threshold of 0.1, we categorized the Yushu yak bulls into distinct lineages. A total of 18 Yushu yaks were classified into 14 lineages (Figure 5C).

### 2.4. Analysis of Genetic Structure in Yushu Yak Population

**Figure 6:**
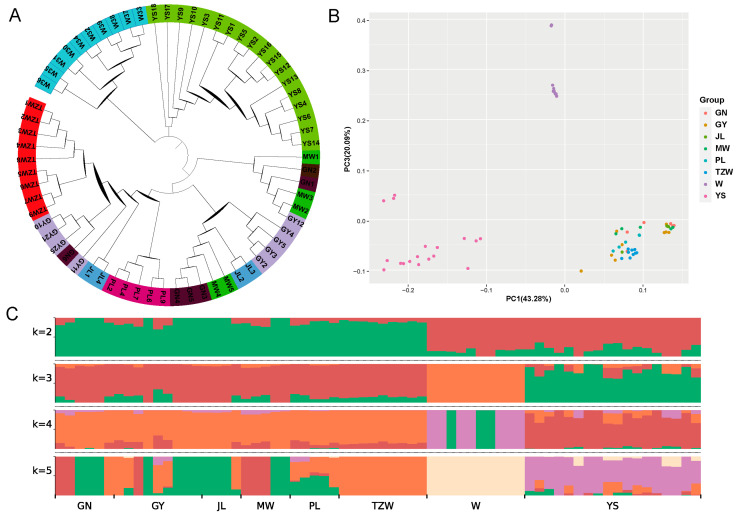
Population structure analysis. (**A**) Phylogenetic tree. (**B**) Principal component analysis. (**C**) Ancestry component analysis (GN: Gannan yak, GY: Plateau yak, JL: Jiulong yak, MW: Maiwa yak, PL: Pari yak, TZW: Tianzhu white yak, W: wild yak, YS: Yushu yak).

The phylogenetic tree reveals that most breeds can be categorized into distinct groups, although a few individuals exhibit signs of population admixture (Figure 6A). The results of the PCA indicate that domestic yaks cluster together as one group, wild yaks form another, while Yushu yaks are distinctly classified as a separate group (Figure 6B). The ancestry component analysis shows that at K = 2; the lineage of all breeds is divided into domestic and wild yaks. Gannan yaks, plateau yaks, Jiulong yaks, Maiwa yaks, Tianzhu white yaks, and Pali yaks share a common ancestry, while Yushu yaks exhibit a lineage closely related to wild yaks (Figure 6C). At K = 3, Yushu yaks and wild yaks are clearly differentiated, with all populations classified into Yushu yaks, wild yaks, and other domestic yaks. The Yushu yak population contains components from both domestic and wild yaks, highlighting its unique genetic background compared to other yak breeds. The specific lineage composition consists of unique lineage (82.22%), domestic yaks (10.91%), and wild yaks (6.87%).

### 2.5. Analysis of Whole-Genome Selection Signals in Yushu Yaks

**Figure 7:**
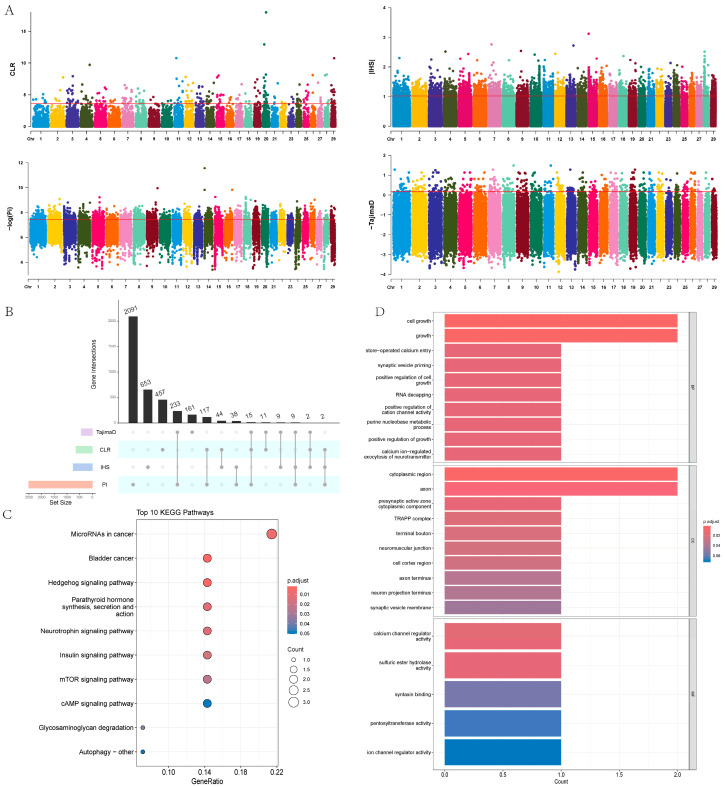
Selection signal analysis. (**A**) Manhattan plots of the four selection methods: π, CLR, IHS, and Tajima’s D. (**B**) UPSET plot combining the four selection methods: π, CLR, IHS, and Tajima’s D. (**C**) KEGG analysis of candidate genes. (**D**) GO enrichment analysis of candidate genes.

In this study, we used four methods—π, CLR, IHS, and Tajima’s D—to detect selection signals within a population of 18 Yushu yaks. The results showed that CLR identified 648, IHS identified 757, π identified 2505, and Tajima’s D identified 440 potential positively selected genes (Figure 7A,B, Table S8). To ensure the reliability of the results, we performed an intersection analysis on genes identified by at least three methods, ultimately identifying 28 candidate genes. We further conducted pathway enrichment analysis on these candidate genes (Figure 7C, Table S9), revealing significant enrichment in pathways such as the Hedgehog signaling pathway, parathyroid hormone synthesis, secretion and action, insulin signaling pathway, mTOR signaling pathway, glycosaminoglycan degradation, and autophagy pathways. Functional enrichment analysis indicated that these candidate genes mainly play crucial roles in biological processes and cellular components (Figure 7D, Table S10). The enrichment results showed that the candidate genes are primarily involved in cell growth, the purine nucleobase metabolic process, neuromuscular junction development, positive regulation of cell growth, developmental cell growth, and regulation of calcium ion transport.

## 3. Discussion

During the process of evolution, yaks have developed distinctive genetic resources under long-term natural selection. The Yushu yak is a distinctive highland cattle breed found in China, capable of surviving in extremely cold and high-altitude regions. It possesses strong physical endurance and adaptability, making it an essential resource for traditional pastoralism in the northwest of China. However, information regarding the genetic structure, population relationships, and the origin and evolutionary process of Yushu yaks is limited. Therefore, we performed whole-genome sequencing on the Yushu yak population and analyzed their intra-population kinship, genetic parameters, and origins and evolution.

The genetic diversity assessment of Yushu yaks revealed that the Yushu yak population has a higher number of short ROH segments and a lower number of long ROH segments. Further statistics showed that the observed heterozygosity in the Yushu yak population is higher than expected heterozygosity. This indicates that the Yushu yak has high genetic diversity and rich in genetic polymorphism, contributing to its strong adaptability to environmental changes. The length of ROH segments is related to the degree of kinship between individuals and their common ancestors; longer ROH segments indicate closer kinship [17]. Inbreeding depression is a significant barrier to species evolution, and assessing kinship within populations can more accurately measure the extent of inbreeding depression, laying the foundation for quality breeding programs [18]. The detection of ROH and low inbreeding coefficients indicate that the inbreeding level in the Yushu yak population is relatively low, which may help maintain genetic health. This is crucial for the adaptability and long-term survival of the population. The genetic background and family structure assessment of Yushu yak breeding bulls found that most individuals have distant kinship, while a few individuals exhibit closer kinship. Using a kinship coefficient exceeding 0.1 as the standard, we classified the Yushu yak breeding bulls into family lineages. After dividing the 18 Yushu yak breeding bulls into 14 family lineages, reasonable family classification can help reduce gene pool limitations and avoid genetic defects caused by inbreeding. This is significant for improving the breeding efficiency of Yushu yaks.

The population structure analysis of Yushu yaks showed that at K = 2, Yushu yaks share a similar genetic background with wild yaks; at K = 3, Yushu yaks and wild yaks are distinguished from each other. Meanwhile, PCA results also indicated that all populations can be divided into three parts: domestic yaks, wild yaks, and Yushu yaks. Earlier genomic analyses have shown that Chinese yaks are mainly divided into two major groups, wild yaks and domestic yaks [3], which aligns with our findings. Previous studies have conducted phylogenetic analyses to compare Jinchuan yaks and Subei yaks with wild yaks as well as other domesticated yak breeds. These studies revealed that Jinchuan yaks and Subei yaks form distinct groups within the population of Chinese yaks. They show significant differences from other domestic yak breeds [14,19]. In our study, Yushu yaks were classified as a distinct subgroup within domesticated yaks, indicating that their genetic background is relatively unique compared to other domestic yak breeds. This difference may be due to geographic isolation or the introduction of wild yak genes. Nevertheless, the genetic background of Yushu yaks still includes a small proportion of domestic yak (10.91%) and wild yak (6.87%) components. This might be due to long-term grazing traditions leading to gene flow between this breed and surrounding yak populations. Our results indicate that Yushu yaks exhibit significant genetic differences from other domestic yak breeds in terms of genetic evolution.

After detecting selection signals within the Yushu yak population, GO enrichment analysis of genes annotated in positively selected regions showed significant enrichment in biological processes related to cell growth and development, metabolic homeostasis, and ion dynamic balance. Among these, the *NRN1L* and *RPTOR* genes were specifically enriched in terms such as “positive regulation of cell growth” and “cell growth”. *NRN1L* (Neurogenin 1-like protein) belongs to the *NRN1* (Neuroregulin 1) family, whose members are involved in regulating cellular signaling, growth, and differentiation. Recent genome-wide association studies have found that *NRN1L* is significantly associated with feed efficiency in goats [20], suggesting its potential role in energy metabolism. *RPTOR* (Regulatory Associated Protein of mTOR) is a key component of the mTOR signaling pathway, which controls cell growth and metabolism by regulating processes such as protein synthesis [21,22]. Studies have shown that mTORC1 regulates skeletal development by mediating mRNA translation during the differentiation stage of osteoblast precursors, and the absence of *RPTOR* can lead to reduced bone mass accumulation [22,23,24]. Additionally, two genes closely related to cardiac muscle development, *STIM1* and *FOXP1*, were identified among the positively selected genes. *STIM1* maintains heart function by mediating smooth muscle contraction, and cardiomyocyte-specific knockout of *STIM1* leads to disorders in glucose and lipid metabolism [25,26]. *FOXP1*, as a transcription factor, regulates heart morphogenesis and functional maintenance [27,28], and recent studies have revealed that its overexpression can significantly increase glucose uptake rates and lactate production levels in fat and muscle cells [29]. The positive selection of these genes may drive the growth and developmental advantages of Yushu yaks in extreme high-altitude environments.

KEGG enrichment analysis of positively selected genes in Yushu yaks showed significant clustering in the insulin signaling pathway and the mTOR signaling pathway. The insulin signaling pathway regulates energy metabolism by coordinating processes such as glucose utilization and protein synthesis [30,31]. Its enrichment may reflect the Yushu yak’s efficient energy acquisition capability in cold, high-altitude environments, thereby supporting their large body size. The mTOR signaling pathway, a core regulatory network for cellular metabolism and growth, integrates nutrient and energy signals to dynamically balance biosynthesis and autophagy [32,33]. Notably, the positively selected gene *CLUH* was found to be involved in energy metabolism regulation. This gene maintains ATP homeostasis by binding to mitochondrial mRNA [34], and its knockout leads to abnormal mitochondrial aggregation, resulting in defects in oxidative phosphorylation and glucose metabolism disorders [35,36]. These results indicate that Yushu yaks exhibit prominent selection features in energy metabolism.

## 4. Materials and Methods

### 4.1. Ethics Statement

All experimental procedures involving cattle were conducted in strict compliance with the Regulations for the Administration of Experimental Animals (approved by the State Council of the People’s Republic of China). This study was formally approved by the Animal Administration and Ethics Committee of Lanzhou Institute of Husbandry and Pharmaceutical Sciences, Chinese Academy of Agricultural Sciences (CAAS) (Permit No.: SYXK-2024-0023). Additionally, the study adheres to the guidelines outlined in the ARRIVE Checklist to ensure transparency and rigor in reporting. All protocols, including sample collection procedures, were executed with explicit authorization from the animal owners and under the supervision of institutional ethics standards.

### 4.2. Sampling and Blood Genomic Extraction

To study the whole-genome selection characteristics of Yushu yaks, we collected blood samples from the jugular veins of 18 male Yushu yaks aged 2–4 years in and around the Yushu Tibetan Autonomous Prefecture of Qinghai Province. During the blood collection process, EDTA was used as an anticoagulant to ensure that the blood samples would not coagulate during transportation and storage. The samples were then stored in a −80 °C freezer. Genomic DNA was extracted using the standard phenol–chloroform method and subsequently subjected to quality assessment. The samples were then sent to Kangsheng Xuyuan Biotechnology Co., Ltd. (Wuhan, China) for whole-genome resequencing. Additionally, we gathered 48 supplementary samples from the public database NCBI for whole-genome sequence alignment (Gannan yak n = 6, Plateau yak n = 9, Jiulong yak n = 4, Maiwa yak n = 5, Pari yak n = 5, Tianzhu white yak n = 9, wild yak n = 10) (Table S11).

### 4.3. Whole-Genome Sequencing

Qualified DNA samples were randomly fragmented to a length of 350 bp using a Covaris sonicator (Covaris, Inc., Woburn, MA, USA). The TruSeq Library Construction Kit was employed for library preparation, strictly adhering to the recommended reagents and materials outlined in the protocol. Upon completion of the library construction, initial quantification was conducted using Qubit 3.0, diluting the library to 1 ng/μL. Subsequently, the insert size of the library was assessed using an Agilent 2100 (Agilent Technologies, Santa Clara, CA, USA), and once the insert size met expectations, precise quantification of the library’s effective concentration was performed using Q-PCR (Applied Biosystems (by Thermo Fisher Scientific, Waltham, MA, USA) (with effective concentration > 2 nM) to ensure library quality. Sequencing of the genomic DNA from each individual was conducted on the DNBSEQ-T7 platform, utilizing 150 bp paired-end reads.

### 4.4. Data Quality Control and Alignment

To ensure the quality of the sequencing results, we employed fastp software (v0.24.0) for quality control of the obtained reads, filtering out low-quality sequences [37]. The quality-controlled sequencing reads were then aligned to the yak reference genome using BWA software (v0.7.18) (genome version: Bosgruv3.0, NCBI accession number: GCA005887515.1) [38]. Following alignment, we utilized Samtools software (v1.10) to sort the BAM files, generating sorted BAM files, and employed Sambamba software (v1.0.1) to identify and remove PCR-introduced duplicate reads from the aforementioned BAM files [39,40].

### 4.5. SNP Detection and Annotation

The HaplotypeCaller, GenotypeGVCFs, and SelectVariants modules of GATK (v4.3.0.0) were used for SNP detection. To ensure high-quality SNPs, the VariantFiltration module in GATK was used with the following filtering threshold values for the parameters: quality by depth (QD) < 2.0, quality (QUAL) < 30.0, StrandOddsRatio (SOR) > 3.0, the Phred-scaled probability of strand bias (FS) > 60.0, mapping quality (MQ) < 40.0, MQRankSum < −12.5, and ReadPosRankSum < −8.0 [41]. The SNP sites with a genotype missing rate > 2 were retained using the criteria F_MISSING < 0.1 and MAC > 2 in BCFtools (v1.10.2) [42]. SNPs were annotated using the ANNOVAR software (v4.3.9.5) based on the annotation files of the reference genome [43].

### 4.6. CNV and SV Detection and Annotation

The analysis of whole genome copy number variations (CNVs) in the Yushu yak was conducted using CNVcaller [44]. The specific steps are as follows: (1) the reference genome was segmented into windows of 800 bp to construct a reference genome database; (2) the number of reads and aligned reads for each window in the BAM file were computed; (3) copy number adjustments were performed; (4) GC content was corrected and standardized; (5) genotype determination was executed. Following the acquisition of the initial CNVR set, the allele frequencies of deletions and duplications (AF) were calculated to classify CNVR types: (1) deletion type: 0.05 < AF (deletion) ≤ 1 and AF (duplication) ≤ 0.01; (2) duplication type: 0.05 < AF (duplication) ≤ 1 and AF (deletion) ≤ 0.05; (3) composite type: 0.05 < AF (duplication) < 0.95 and 0.05 < AF (deletion) < 0.95. Subsequently, filtering was performed based on the following criteria: (1) silhouette score > 0.6; (2) filtering according to CNVR length: deletion-type CNVRs not exceeding 50 kb, duplication-type CNVs not exceeding 500 kb, and composite-type CNVRs not exceeding 50 kb. The obtained CNVR set was annotated using ANNOVAR software. Thereafter, the BAM files of all individuals were imported into Manta software (v1.6.0) for direct identification of SVs and genotypes at the population level, with the SV set subsequently annotated using ANNOVAR software [43,45].

### 4.7. Genetic Diversity and ROH Detection

Nucleotide diversity (Pi) was calculated using VCFtools (v0.1.16), with a sliding window of 50 kb and a step size of 20 kb [46]. The Plink (V1.90) software was employed to analyze the effective population size, polymorphic information content, proportion of polymorphic markers, expected heterozygosity, observed heterozygosity, effective allele count, and minimum allele frequency of the Yushu yak based on SNP data [47]. After assessing the ROH on each chromosome using Plink, the inbreeding coefficient for each individual was computed based on the ROH. The formula for calculating FROH is as follows: FROH = sum of ROH segment lengths on autosomes/total length of autosomes. The specific parameters for ROH calculation were: (1) --homozyg-density 50, (2) --homozyg-gap 1000, (3) --homozyg-kb 100, (4) --homozyg-snp 50, (5) --homozyg-window-het 5, (6) --homozyg-window-missing 5, (7) --homozyg-window-snp 50, (8) --homozyg-window-threshold 0.05.

### 4.8. Kinship Analysis and Pedigree Construction

Genetic distances between individuals were calculated using PLINK software (v1.90) to construct an IBS distance matrix [47]. The GCTA software was employed to create a genetic relationship (G) matrix among individuals [48]. Subsequently, R language was utilized for visualization and analysis of kinship among individuals within the population. Based on the IBS distance matrix, a phylogenetic tree was constructed using the Neighbor-Joining (NJ) method via PHYLIP software (v3.6), and visualization was performed using MEGA (v11.0.13) [49,50]. Thereafter, in conjunction with the results of the kinship analysis, families were delineated based on a kinship coefficient of 0.1 or greater, allowing for an examination of the familial structure within the Yushu yak population.

### 4.9. Population Structure and Phylogenetic Tree

After filtering the original SNP results with --maf 0.05 and --geno 0.2, Plink (v1.90) was utilized to remove linkage disequilibrium (--indep-pair-wise 50 5 0.2). PCA analysis was conducted using GCTA software (v1.94.1), and visualizations were created with custom R scripts. To accurately identify the ancestral components of each breed, ADMIXTURE software (v1.3.0) was employed to analyze population structure, setting the number of ancestral populations (K) from 2 to X, with X determined by specific results [51]. The phylogenetic tree was generated by calculating genetic distances with Plink, resulting in a distance matrix file, which was subsequently visualized using MEGA (v11) and ITOL (v7.1) [50,52].

### 4.10. Selection Signal Analysis

To elucidate the adaptive traits of Yushu yaks and their genetic adaptation to the local environment, we employed four methods to detect genomic selection signals in this population. The π, CLR, IHS, and Tajima’s D statistics were utilized to identify selection signals within the Yushu yak population, aiming to filter for distinct adaptive traits. The π and Tajima’s D values were calculated using VCFtools (v0.1.16) within a 50 kb window and a 20 kb step size, while CLR was computed using SweeD software (v3.0) with a 100 kb window [53]. IHS was calculated and standardized using Selscan, with the upstream and downstream 20 kb regions of the top 1% loci subjected to gene annotation [54]. For each method, appropriate thresholds were established, and genes that met the threshold criteria in at least three methods were subjected to overlapping selection. Finally, we conducted online Gene Ontology (GO) and Kyoto Encyclopedia of Genes and Genomes (KEGG) pathway enrichment analyses for the identified candidate genes using KOBAS 3.0 to explore their functional roles and signaling pathways [55].

## 5. Conclusions

This study utilized whole-genome resequencing technology to analyze the genomic sequences of Yushu yaks. The sequencing results revealed numerous breed-specific variant sites, significantly enriching the Chinese yak genome database. The findings indicate that the Yushu yak population exhibits high genetic diversity and low inbreeding coefficients. Population structure analysis showed significant differences between Yushu yaks and other domestic yak populations as well as wild yak populations. Additionally, through selective sweep analysis, we found that positively selected genes in Yushu yaks are significantly enriched in pathways related to cell growth and development, and insulin signaling pathways. These results suggest that Yushu yaks possess notable selection features in growth and development as well as energy metabolism. The findings of this study provide important insights into the population structure and superior genetic traits of Yushu yaks, laying a theoretical foundation for the sustainable utilization of these breed resources and future breeding improvements.

## Figures and Tables

**Table 1:** Parameters related to genetic diversity in Yushu yaks.

Parameter	Minimum Allele Frequency	Polymorphic Marker Ratio	Expected Heterozygosity	Observed Heterozygosity	Nucleotide Diversity
Yushu Yaks	0.2033	0.2869	0.2869	0.3074	0.0018

## Data Availability

The data that support the findings of this study are available from the corresponding author upon reasonable request.

## Supplementary Materials

The following supporting information can be downloaded at https://www.mdpi.com/article/10.3390/ijms26083879/s1.

## Abbreviations

The following abbreviations are used in this manuscript:

SNP	Single nucleotide polymorphism
WGS	Whole-genome sequencing
π	Nucleotide diversity
GO	Gene ontology
KEGG	Kyoto Encyclopedia of Genes and Genomes
NJ	Neighbor-joining
PCA	Principal component analysis
ROH	Runs of homozygosity
CLR	Composite likelihood ratio
HIS	Integrated Haplotype Score
GN	Gannan yak
GY	Plateau yak
JL	Jiulong yak
MW	Maiwa yak
PL	Pari yak
TZW	Tianzhu white yak
W	Wild yak
YS	Yushu yak
